# Brief report: Free-living physical activity levels and cognitive control in multi-problem young adults

**DOI:** 10.3389/fnhum.2022.994123

**Published:** 2022-10-21

**Authors:** Maria Elise van der Sluys, Reshmi Marhe, Peter H. van der Laan, Arne Popma, Erik J. A. Scherder

**Affiliations:** ^1^Department of Clinical, Neuro and Developmental Psychology, Vrije Universiteit Amsterdam, Amsterdam, Netherlands; ^2^Department of Psychology, Education and Child Studies, Erasmus University Rotterdam, Rotterdam, Netherlands; ^3^Department of Criminal Law and Criminology, Vrije Universiteit Amsterdam, Amsterdam, Netherlands; ^4^Netherlands Institute for the Study of Crime and Law Enforcement, Amsterdam, Netherlands; ^5^Department of Child and Adolescent Psychiatry, VU University Medical Center, Amsterdam, Netherlands

**Keywords:** physical activity, cognitive control, multi-problem young adults, brief report, ecologically valid sample

## Abstract

Previous studies indicate a positive association between physical activity and cognitive control in sedentary but healthy adults, yet not much is known about physical activity levels in multi-problem young adults. We examined the level of self-reported free-living physical activity (i.e., MET minutes per week) in an ecologically valid sample of young adults facing multiple problems, including unemployment, lack of education, frequent substance use, and history of delinquency. We compared cognitive control with an age- and sex-matched control sample. Additionally, the association between physical activity and cognitive control (i.e., response inhibition, error processing, interference effect) in the multi-problem group was examined. Physical activity and cognitive control were measured with the International Physical Activity Questionnaire-Long Form and three cognitive control experiments (i.e., Flanker, Go/NoGo, Stroop), respectively. With *M* = 4428 Metabolic Equivalents (METs), our multi-problem sample (*n* = 63) showed physical activity levels similar to the age- and sex-matched control sample from the general population (*n* = 62). The multi-problem young adults also showed impaired cognitive control indexed as decreased response inhibition and decreased Flanker correctness effect compared to their peers. We could not find an association between self-reported physical activity and cognitive control in the multi-problem sample. Due to the small sample size, results should be interpreted with caution. However, future dose-response studies could still use these results to further examine if within-individual increased physical activity may possibly lead to improved cognitive control in (already relatively active) multi-problem young adults.

## Introduction

Cognitive control (also called executive functioning) comprises a set of top-down processes enabling self-regulation, future- and goal-oriented behavior, and the focus of attention (Diamond, 2013). Deficits in cognitive control are found in several clinical disorders, including attention-deficit/hyperactivity disorder, autism spectrum disorder, and traumatic brain injury (Craig et al., 2016). Besides clinical disorders, there is also evidence that basic physical activity levels are associated with altered cognitive control functioning. Results from observational studies show impaired cognitive control in sedentary individuals compared to active individuals across the human lifespan (Hillman et al., 2008; Kamijo et al., 2009; Chaddock et al., 2010; Kamijo and Takeda, 2009, 2010; Bae et al., 2012; Padilla et al., 2013, 2014; Pérez et al., 2014; Verburgh et al., 2014; van der Niet et al., 2015; Baumgartner et al., 2018).

More specifically, in sedentary but otherwise healthy young adults there are indications for impaired cognitive control, reflected in a relative inability to restrain inappropriate responses (i.e., response inhibition) (Hogan et al., 2013), to adequately process errors (i.e., error processing) (Pérez et al., 2014), and to suppress an automated response in favor of a less automated response (i.e., cognitive interference) (Giles et al., 2017; Goenarjo et al., 2020). Not surprisingly, the same cognitive control deficits (i.e., impaired response inhibition, error processing, and cognitive interference) have been found in individuals displaying antisocial behavior including aggression, delinquency, and substance use (Hiatt et al., 2004; Swann et al., 2009; Zeier et al., 2012; Marhe et al., 2013; Weidacker et al., 2017; Turner et al., 2018). These similarities are exemplified in task performance on a Flanker task (a reaction-time task that is frequently used to measure error processing indices), with significantly lower Flanker accuracy in low active preadolescents (*M* = 88.6, *SD* = 2.4) compared to medium active (*M* = 96.1, *SD* = 1.8) and high active (*M* = 98.4, *SD* = 0.2) preadolescents in the general population (Zhu et al., 2022). Like low active youngsters, low Flanker accuracy is found in substance abusers (*M* = 84) vs. non-users (*M* = 91) (Marhe et al., 2013) and in multi-problem young adults (*M* = 81, *SD* = 0.16) vs. controls from the general population (*M* = 87, *SD* = 0.09) (Zijlmans et al., 2019). Although sedentary and problem populations show similar results on cognitive control measures, it is not yet known whether the physical activity level plays a role in the diminished cognitive control processing of multi-problem young adults.

Previous studies uncovered a positive association between physical activity and cognitive control in healthy young adults from the general population, with higher physical activity levels associated with better cognitive control (Hogan et al., 2013; Pérez et al., 2014; Giles et al., 2017; Goenarjo et al., 2020). Moreover, there is ample evidence that an increase in physical activity enhances cognitive control (Colcombe and Kramer, 2003; Hillman et al., 2008; Smith et al., 2010; Gomez-Pinilla and Hillman, 2013; Guiney and Machado, 2013; Verburgh et al., 2014), particularly in individuals with a sedentary lifestyle (Gomez-Pinilla and Hillman, 2013; Verburgh et al., 2014). Ultimately, if physical activity can enhance cognitive control, it might help populations that are compromised in their cognitive control functioning. As multi-problem young adults are such a population, they might benefit from extra physical activity. However, as a first step, it is important to gain knowledge on the level of physical activity, i.e., the art of lifestyle, and the association with cognitive control in this group of young adults.

Physical activity has multiple associated health benefits (Paluska and Schwenk, 2000; Lubans et al., 2012; Daskalopoulou et al., 2017) including reduced risk on physical disorders (e.g., cardiovascular disease and obesity: Carnethon et al., 2003) and reduced risk on mental disorders (e.g., depression and anxiety: Kantomaa et al., 2008; Rebar et al., 2015). According to the American College and Sports Medicine (ACSM) and the World Health Organization (WHO), (young) adults should achieve at least 150 min of moderate physical activity, or 60 (ACSM) to (WHO) of vigorous-intensity physical activity per week (Riebe et al., 2018; Bull et al., 2020; World Health Organization [WHO], 2020). Most young adults from the general population do not meet these recommendations (Song et al., 2013; Marques et al., 2015). To the authors’ knowledge, it is unknown if these recommendations are met by young adults facing multiple problems including a lack of daytime activities (such as work or education), low or no income, behavioral and psychological problems, frequent substance use, and a history of delinquency (Luijks et al., 2017; van Duin et al., 2017, 2018; Zijlmans et al., 2018, 2019, 2020a,b; van der Sluys et al., 2020). Previous research on physical activity levels in these or similar populations is lacking or prison-based (Fischer et al., 2012) and related research on factors associated with level of physical activity in such populations indicates mixed results. For example, having parents without a high school diploma (Singh et al., 2008) and having a low socio-economic status (SES) (Stalsberg and Pedersen, 2010) are associated with a sedentary lifestyle, yet lack of means of transportation (Spinney and Millward, 2010), e.g., due to low or no income, is associated with a more active lifestyle. Thus, more information is needed on physical activity levels in young adults displaying multiple problems (including antisocial behavior).

The current study therefore first aims to examine the self-reported free-living (i.e., habitual) physical activity in multi-problem young men aged 18–27 (Luijks et al., 2017; van Duin et al., 2017, 2018; Zijlmans et al., 2018, 2019, 2020a,b; van der Sluys et al., 2020). Due to mixed results in previous literature (Singh et al., 2008; Spinney and Millward, 2010; Stalsberg and Pedersen, 2010), it is not possible to form a concrete hypothesis on the level of physical activity. To confirm the expected impairment in cognitive control as measured with the behavioral measures of response inhibition, error processing, and cognitive interference (Hiatt et al., 2004; Swann et al., 2009; Zeier et al., 2012; Marhe et al., 2013; Weidacker et al., 2017; Turner et al., 2018; Zijlmans et al., 2020a), we perform a supplementary analysis comparing the multi-problem young adults with age- and sex matched controls from the general population. Second, we examine if there is a possible association between free-living physical activity and cognitive control. Based on literature in healthy young adults from the general population, we expect a positive association in the multi-problem group (Hogan et al., 2013; Pérez et al., 2014; Giles et al., 2017; Goenarjo et al., 2020).

## Materials and methods

### Participants

Participants were 63 multi-problem young adult men aged 18-27, recruited at the start of the day treatment program *De Nieuwe Kans* (DNK; translated as “New Opportunities,” for more information on the program (see Luijks et al., 2017; van der Sluys et al., 2020). In short, DNK offers practical support and cognitive behavioral therapy to young adult men (aged 18–27) suffering from a range of problems including low or no income, low or no education, lack of daytime activities, frequent drug use, and a criminal record. The main aim of the treatment is to increase self-sufficiency through education and employment. Fifteen participants were excluded from the analyses due to failure to complete the cognitive control measures (*n* = 13) or questionnaires (*n* = 2). Thus, the final sample of multi-problem young adults included (*n* = 50) for the comparison on cognitive control with age- and sex matched controls from the general population (*n* = 62, mean age 23.6, *SD* 2.7) and (*n* = 48) for the analysis on the association between physical activity and cognitive control. In addition, to confirm if our multi-problem sample suffered from more problems on various other secondary outcomes measured with questionnaires, multiple independent sample *t*-tests were run (*n* = 61). The multi-problem young adults experienced significantly more problems, showing less education, overall less daytime activities, overall more family problems, more impulsivity, and more years of regular cannabis use compared to the control sample (see Supplementary Table 1). Surprisingly, alcohol consumption was larger in the control sample, possibly because this group comprises mostly college students where high rates of (binge) drinking are associated with multiple individual and environmental changes (Krieger et al., 2018).

*A priori* sample size calculations indicated a required sample size of *N* = 86 for the comparison between groups on cognitive control (power = 0.80, *f*^2^ = 0.25: Morgan and Lilienfeld, 2000) and *N* = 92 for the regression on physical activity and cognitive control within the multi-problem group (power = 0.80, *f*^2^ = 0.15: Cameron et al., 2015). However, due to the unforeseen closing of the day treatment program as a direct result of the COVID-19 pandemic, it was not possible to continue data collection.

### Cognitive control

All cognitive control experiments (Flanker, Go/NoGo, Stroop) were self-administered with E-Prime 3.0 software (Stoet, 2017, 2010) after an explanation from a trained researcher.

Response inhibition was measured with a Go/NoGo task previously described in Luijten et al. (2013) and Zijlmans et al. (2020a). To summarize, participants were required to press the spacebar with their left or right hand on a QWERTY-keyboard in response to a letter (Go trials) and withhold this response when the presented letter was a repetition of the previous one (NoGo trials). Stimuli were presented at 1 HZ and shown for 700 ms, followed by a blank screen for 300 ms. The total response window was set at 1000 ms (700 ms + 300 ms). A total of 817 Go and 110 NoGo trials (i.e., 12% NoGo trials) were presented in an unpredictable manner by introducing jitter in the number of intervening Go trials (*M* = 7.25, range 3–16). The task included four short rest moments (15 seconds each). Accuracy on the NoGo trials was used as measure for response inhibition with higher accuracy on the NoGo trials indicating better response inhibition.

Error processing was measured with a Flanker task previously described in Zijlmans et al. (2019). In short, participants were shown congruent and incongruent letter strings (HHHHH, SSSSS, HHSHH, SSHSS). They were required to respond to the middle letter by pressing the corresponding letter on a QWERTY-keyboard with their left or right index finger as fast and accurate as possible. The task consisted of 200 congruent and 200 incongruent trials, presented at random. Each letter string was presented for 52 ms followed by a blank screen (648 ms). The total response window was set at 700 ms (52 ms + 648 ms) followed by an inter-stimulus-interval (ISI) of 1000 ms. The task was divided into five blocks of 80 trials with a short pause in between (15 seconds rest). Total accuracy, post error slowing (post error reaction time - post correct reaction time), and correctness effect (reaction time correct trials - reaction time incorrect trials) were calculated as response measures for the Flanker task. Higher post-error slowing is interpreted as better error processing.

Lastly, interference or Stroop effect was measured with a computerized non-verbal Stroop Color-Word Test. Participants were presented with a word (red, blue, yellow, green) written in one of the same colors (red, blue, yellow, green). They were instructed to respond to the color of the ink by pressing the letters d, f, j, or k, representative of a matching color shown on screen. Participants were asked to respond as fast and accurate as possible. The trial started with a fixation cross shown for 250 ms, followed by the colored word which was shown for 3000 ms or until the respondent responded. After that, the feedback followed (“correct,” “incorrect,” and “no response detected”) which was shown for 500 ms. During the practice trials, participants were shown a colored letter string (XXXXX) in one of the colors to practice the button press. The experiment with the colored words consisted out of two similar blocks divided by a self-paced rest period. Participants were shown a total of 192 trials, divided into 144 incongruent (different written word and color) and 48 congruent trials (same written word and color), prefaced by 40 practice trials (i.e., 30 congruent and 10 incongruent trials). The interference effect (reaction time incongruent trials - reaction time congruent trials) was calculated and used as an outcome measure. A higher score is interpreted as increased cognitive interference.

### Questionnaires

All questionnaires were asked in the form of a structured interview using Castor Electronic Data Capture (IBM, 2011). Free-living physical activity was measured with the long version of the International Physical Activity Questionnaire (IPAQ: Hagströmer et al., 2006). The IPAQ is a self-report questionnaire for adults (aged 18–65) measuring the frequency, duration, and intensity of physical activity over the past 7 days. Total physical activity expressed as Metabolic Equivalents (METs) minutes per week was used as measure of physical activity level. One MET represents an activity burning 1 kcal per kilogram bodyweight per hour. MET minutes per week are calculated by multiplying weekly duration per activity (daily activity × number of days of performed activity) by the MET values corresponding to the required exertion for that specific activity (Ainsworth et al., 2000).

Because the association between physical activity and cognitive control may be influenced by body composition (Galioto Wiedemann et al., 2014) and intelligence (Hillman et al., 2005), we included Body Mass Index (BMI) and the score on a screener for low intelligence or a learning disability as control variables. BMI was measured by dividing self-reported height^2^ by self-reported weight. BMI is an international measurement used for classifying adults based on body weight and height. Lastly, a Screener for Intelligence and Learning Disabilities (SCIL 18+; Nijman et al., 2018) was used to screen for mild to borderline intellectual disabilities. The total score on the screener was included. A score of 19 or lower is indicative of possible intellectual disabilities.

### Procedure

This study was approved by the Scientific and Ethical Review Board of the VU University Amsterdam (VCWE-2017-139). Participants provided written informed consent prior to study participation. A reimbursement of 10 euros was distributed for completing the questionnaires and cognitive control-tasks.

**FIGURE 1:**
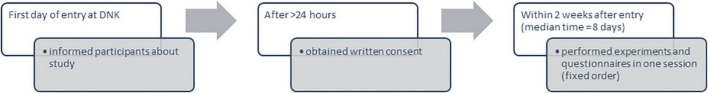
Timeline of recruitment and testing.

On the first day of entry at DNK, all treatment-seekers at DNK were informed about the content of the study. They were given at least 24 hours to decide about their participation. After giving written consent, participants were tested within 2 weeks after entry at DNK by trained researchers (median time between testing and entry: 8 days) at DNK. The cognitive control experiments and questionnaires were tested in the same session, starting with an explanation, then the experiments in fixed order, and ending with the questionnaires (see Figure 1). Participants were eligible if they had sufficient knowledge of the Dutch language and did not suffer from colorblindness.

Participants for the control sample were recruited through university message boards or *via* social media such as Facebook or Instagram, using snowball sampling and convenience sampling. Participants were eligible for the control sample if they were male, aged between 18 and 27, had sufficient knowledge of the Dutch language, and did not suffer from colorblindness.

### Statistical analyses

IBM SPSS Statistics for Windows version 20.0 (IBM, 2011) was used to perform the statistical analyses. First, self-reported free-living physical activity was examined by reporting the continuous MET score of the IPAQ. Additionally, to examine if the multi-problem sample (*n* = 50) vs. age-sex matched controls (*n* = 62; between-group variable) showed the expected impairment on the selected measures of response inhibition, error processing, and cognitive interference (i.e., five dependent variables), a one-way multivariate analysis of variance (MANOVA) was performed. For the MANOVA, data was bootstrapped due to non-normality of NoGo accuracy. Bonferroni correction was applied to correct for multiple testing in the univariate tests (*p* = 0.01). Lastly, within the multi-problem sample (*n* = 48), the association between free-living physical activity (i.e., MET score; dependent variable) and the selected measures of response inhibition, error processing, and cognitive interference (i.e., five predictor variables) was tested with an exploratory hierarchical linear regression analysis with two steps. The continuous score on the SCIL and BMI were entered as covariates in the first step with the cognitive control measurements added in the second step. Due to violation of the normality of residuals assumption, the log (LN) transformation of the MET score was computed and used in the regression. This solved the non-normality of the residuals. None of the other assumptions was violated. The level of significance was set at *p* = 0.05 for the linear regression.

## Results

### Self-reported free-living physical activity

The multi-problem young adults scored *M* = 4428 MET minutes per week (Table 1).

**TABLE 1:** Main outcome measures of multi-problem young adults and healthy young adults from the general population.

	Multi-problem sample (*N* = 50)		Control sample (*N* = 62)		Univariate testing	Between group effect size
				
Variable	*M*	*SD*	*M*	*SD*	*p*	Cohen’s *d*
Stroop interference (RT incongruent—RT congruent)	75.94	64.04	61.26	46.97	0.165	0.26
Flanker total accuracy	0.81	0.16	0.86	0.08	0.030	–0.39
Flanker post-error slowing (RT post error—RT post correct)	26.03	59.58	15.50	40.34	0.269	0.20
Flanker correctness effect (RT correct—RT incorrect)*	–28.36	75.99	12.71	42.62	<0.001	–0.66
NoGo accuracy*	0.52	0.17	0.68	0.11	<0.001	–1.11
IPAQ METs	4428.74	4259.26	4853.67	3978.42		–0.10
BMI (weight in kg/height in cm^2^)	22.83	4.11	23.30	3.15		–0.12
SCIL intelligence total	13.58	5.36				

IPAQ, International Physical Activity Questionnaire; MET, Metabolic Equivalents minutes per week; BMI, Body Mass Index; SCIL, Screener for Intelligence and Learning Disabilities. Cohen’s *d* = ((*M*_multi–problem_ – *M*_control sample_)/*SD*_pooled_).

*p < 0.01 (Bonferroni correction applied).

### Cognitive control

Results from the MANOVA indicate significant cognitive control deficits in response inhibition and performance on a Flanker task in the multi-problem sample compared to the control group [*F*(5, 106) = 13.81; *p* < 0.01; partial η^2^ = 0.983], specifically for NoGo accuracy [*F*(1, 110) = 32.68; *p* < 0.001; partial η^2^ = 0.22], and Flanker correctness effect [*F*(1, 110) = 13.05; *p* < 0.001; partial η^2^ = 0.10]. See Table 1 for side-by-side comparison of means.

In the explorative linear analysis within the multi-problem group, we did not find the hypothesized significant association between free-living physical activity (i.e., MET minutes per week) and cognitive control (i.e., Stroop interference, Flanker total accuracy, Flanker post-error slowing, Flanker correctness effect, NoGo accuracy; Table 2). The first model containing only the covariates (SCIL, BMI) was not significant (*p* = 0.849, adj *R*^2^ = –0.037), nor was the model with the added cognitive control measures (*p* = 0.225, adj *R*^2^ = 0.059). Results should be interpreted with caution due to the small sample size.

**TABLE 2:** Results of linear regression examining free-living physical activity and cognitive control in multi-problem young adults (*N* = 48).

					95% CI B	
					
	B	SE (*B*)	*p*	*t*	Lower	Upper
**Model 1**						
Constant	8.235	0.901	<0.001	9.140	6.420	10.050
BMI (weight in kg/height in cm^2^)	–0.003	0.036	0.941	–0.075	–0.075	0.069
SCIL	–0.016	0.027	0.575	–0.565	–0.071	0.040
**Model 2**						
Constant	7.177	1.143	<0.001	6.281	4.867	9.486
BMI (weight in kg/height in cm^2^)	0.000	0.035	0.997	–0.004	–0.071	0.071
SCIL	–0.047	0.030	0.124	–1.573	–0.107	0.013
Stroop interference (RT incongruent—RT congruent)	–0.001	0.003	0.821	–0.227	–0.006	0.005
Flanker total accuracy	1.360	1.324	0.311	1.027	–1.317	4.036
Flanker post-error slowing (RT post error—RT post correct)	0.007	0.004	0.070	1.858	–0.001	0.014
Flanker correctness effect (RT correct—RT incorrect)	0.004	0.003	0.176	1.377	–0.002	0.011
NoGo accuracy	0.593	1.027	0.567	0.577	–1.484	2.669

BMI, Body Mass Index; SCIL, Screener for Intelligence and Learning Disabilities.

## Discussion

Although the level of physical activity (4428 MET) in the multi-problem young adult group was well above adult WHO recommendations (900 MET: (World Health Organization [WHO], 2020), these levels are comparable with the physical activity in the age- and sex matched control sample (*M* = 4853). Similarly, in a previous study with Ukrainian students using the same IPAQ questionnaire as the current study, total mean activity was 3560 MET (Bergier et al., 2014). This may indicate that (multi-problem) young adults are more active than previously expected based on adolescent (Song et al., 2013) and adult (Marques et al., 2015) samples not suffering from multiple problems, which mostly do not meet these recommendations. More specifically, there is a global trend related to low levels of physical activity among adolescents and (young) adults from the general population (Valle et al., 2015; Guthold et al., 2020), with physical activity levels further declining between adolescence and young adulthood (for a meta-review, see Corder et al., 2019). Less is known about individuals suffering from a range of (psychological, financial, and behavioral) problems such as the current multi-problem sample, although there are indications for a sedentary lifestyle in similar populations (such as having parents without a high school diploma (Singh et al., 2008), a low SES (Stalsberg and Pedersen, 2010), and having only one parent present (Junger et al., 2001). As the current results indicate high physical activity levels in multi-problem young adults, contrary to existing research, it may be worthwhile to conduct more studies in young adults displaying several problems. Possible other relevant factors to include in future studies are gender [with males being more active (Bergier et al., 2014, 2017)], lack of having a (sitting) job (Thorp et al., 2012), and place of residence (Bergier et al., 2016).

Consistent with our first hypothesis, we confirmed the cognitive control impairments in our sample of multi-problem young adult men compared to the age- and sex-matched controls from the general population (Hiatt et al., 2004; Swann et al., 2009; Zeier et al., 2012; Marhe et al., 2013; Weidacker et al., 2017; Turner et al., 2018), specifically on response inhibition and the Flanker correctness effect. We also saw a trend for accuracy on the Flanker task, however, after correcting for multiple testing, this result did not remain significant. Effect sizes (Cohen’s *d)* were, respectively, large, small-medium, and medium-large in favor of the control group (Cohen, 1988), which is in line with meta-analytic results on cognitive control in antisocial populations where medium (*d* = 0.44; Ogilvie et al., 2011) and large (*d* = 0.62: Morgan and Lilienfeld, 2000) effect sizes were found in favor of healthy control samples. The current results are similar to other studies in populations showing multiple problems including antisocial behavior and addiction (Swann et al., 2009; Ogilvie et al., 2011; Zeier et al., 2012; Marhe et al., 2013; Weidacker et al., 2017; Turner et al., 2018). Cognitive control may be impaired due to co-existing disorders such as Attention-Deficit/Hyperactivity Disorder (ADHD) or autism spectrum disorder (Craig et al., 2016) or due to traumatic brain injury (Schretlen and Shapiro, 2003), which may be overrepresented in our sample of multi-problem young adults (Storebø and Simonsen, 2016; Bellesi et al., 2019; Chester et al., 2022). Another possible explanation may be the relatively lower IQ in the multi-problem sample compared to their peers from the general population, as previous studies indicate a positive association between IQ and cognitive control (Zijlmans et al., 2019).

However, we could not find support for our second hypothesis, as our results do not indicate a positive association between physical activity and cognitive control in multi-problem young adult men. There was a borderline significant association between post-error slowing and free-living physical activity, but due to the small sample size, it is not possible to draw any definite conclusions. Comparison with existing literature is further complicated as they focus on healthy young adults from the general population (Kamijo and Takeda, 2010, 2009; Padilla et al., 2014, 2013; Pérez et al., 2014; Ho et al., 2018; Salas-Gomez et al., 2020). In accordance with our results, a previous study could also not find a significant effect of physical activity level (low, moderate, high) measured with the IPAQ on a combined cued reaction time task and Flanker task in healthy young adult men and women. More specifically, this association remained non-significant even after reducing task complexity, reducing testing time, and increasing the cognitive load (Ho et al., 2018). Another study using the short version of the IPAQ did find a significant association between total amount of physical activity (measured with MET minutes) and the Stroop task, but not with the Trail-Making Task, and only in young adult woman, but not in men (Salas-Gomez et al., 2020).

The current non-significant findings may be due to the relatively high levels of physical activity in the multi-problem sample. Intervention studies predominantly focus on sedentary individuals, as there is converging evidence that sedentary individuals show impaired cognitive control compared to active individuals (Colcombe and Kramer, 2003; Kamijo and Takeda, 2010, 2009) and thus would benefit the most from enhanced cognitive control through increased physical activity. However, these studies mostly rely on the WHO-guidelines to distinguish between sedentary and active individuals. It has been recently proposed that most health gains occur at 3000–4000 MET minutes per week rather than the recommended 900 MET, with significantly lower risk on multiple diseases including colon cancer and diabetes (Kyu et al., 2016). It may be that the current recommendations are too stringent to distinguish between active and non-active young adults and thus it may still be relevant for future studies to examine if increased physical activity could lead to increased cognitive control in multi-problem young adults. Lastly, it may be possible that other non-accounted for factors mediate or moderate the association between physical activity and cognitive control, such as structural brain abnormalities (Leshem, 2020) and maturation rate of the prefrontal cortex (PFC), an area important for cognitive control (Dahl, 2004; Veroude et al., 2013). Future studies should try to replicate these results, taking our current limitations into consideration.

The present study is not without limitations, as the small sample size (due to failure to complete all outcome measures and due to the COVID-19 pandemic) limits the interpretation of results, specifically in the testing of the association between physical activity and cognitive control. Also, regarding the Flanker task, it is possible that the time window and specifically the ISI was too short to elicit proper post-error slowing results. Although post-error slowing has been found in a similar sample with the same task duration (Zijlmans et al., 2019), a meta-analysis comparing post error slowing in ADHD patients vs. healthy participants showed that a larger ISI resulted in better post-error slowing in the controls than a smaller ISI (Balogh and Czobor, 2016). However, considering the exploratory aim of this study and the lack of research in an ecologically valid sample of multi-problem young adults, the current results could still provide us with valuable insights for future studies. For example, can increased physical activity in this (already active) sample still result in improved cognitive control, similar to results found in other (mostly sedentary) populations and age-groups (Hillman et al., 2008; Chaddock et al., 2010; Verburgh et al., 2014)?

In conclusion, our study is the first to examine self-reported free-living physical activity indexed by MET minutes per week in young adults facing multiple problems. Results indicate comparable levels of self-reported free-living physical activity as an age- and sex matched control sample, similar to other literature in students (Bergier et al., 2014) yet impaired cognitive control (measured with behavioral measures of error processing, response inhibition, and interference) relative to the controls. Despite existing limitations, the present study is the first to explore the association between physical activity and cognitive control in a multi-problem sample suffering cognitive control deficits, providing a starting point for future studies on this topic.

## Data availability statement

The raw data supporting the conclusions of this article will be made available by the authors, without undue reservation.

## Ethics statement

This study involving human participants was reviewed and approved by the Scientific and Ethical Review Board of the VU University Amsterdam. The participants provided their written informed consent to participate in this study.

## Author contributions

MES devised the project, collected the data, and analyzed the data. RM and ES supervised the data. MES wrote the manuscript in consultation with RM, PL, AP, and ES. All authors contributed to the article and approved the submitted version.
