# Supplementary material for: Brief report: Free-living physical activity levels and cognitive control in multi-problem young adults
Source: Front Hum Neurosci. 2022 Oct 21;16:994123. doi: 10.3389/fnhum.2022.994123 (PMC9634251; doi:10.3389/fnhum.2022.994123)
Supplement: Supplementary file 1 [file Table_1.docx]

**Supplementary material**

| **Table S1.** Characteristics of multi-problem young adults and young adults from the general population | | |
| --- | --- | --- |
|  | Multi-problem sample (N = 61) | Age and sex-matched control sample (N = 62) |
| Variable | *M* (*SD*) / % | *M* (*SD*) / % |
| Age | 21.18 (2.77) | 23.63 (2.71) |
| Highest finished education ** |  |  |
| No education | 11.3% | 0% |
| Primary education | 24.2% | 0% |
| Secondary pre-vocational education | 29.1% | 2.5% |
| Secondary senior education | 3.2% | 47.5% |
| Secondary vocational education | 29.0% | 9.5% |
| Higher education | 0% | 40.0% |
| Other | 3.2% | 0% |
| Ethnicity |  |  |
| Western | 19.4% | 82.5% |
| Dutch Antilles | 29.0% | 0% |
| Moroccan | 16.1% | 5.0% |
| Surinamese | 8.1% | 0% |
| African | 8.1% | 1.3% |
| Cape Verdean | 4.8% | 0% |
| Syrian | 3.2% | 2.5% |
| Other | 11.3% | 8.8% |
| Daytime activities |  |  |
| No daytime activities ** | 45.0% | 3.7% |
| Work ** | 20.0% | 70.7% |
| Education ** | 5.0% | 58.5% |
| Care for others | 3.3% | 0% |
| Other ** | 26.7% | 7.3% |
| Family problems in youth |  |  |
| No problems ** | 38.7% | 17.1% |
| One parent (mostly) absent * | 21.0% | 7.3% |
| Mental health problems of family members | 4.8% | 4.9% |
| Domestic violence * | 14.5% | 2.4% |
| Police contact of family members ** | 27.4% | 0% |
| Drug use of family members * | 12.9% | 0% |
| Alcohol use of family members * | 12.9% | 1.2% |
| Other * | 17.7% | 2.4% |
| BSSS sensation seeking total | 24.66 (4.83) | 25.53 (6.08) |
| BIS-11 impulsivity total ** | 68.02 (9.44) | 59.76 (8.73) |
| Alcohol and cannabis use |  |  |
| Cannabis use past 30 days (unit) ** | 0.55 (0.50) | 0.15 (0.35) |
| Alcohol use past 30 days (unit) ** | 0.26 (0.44) | 0.63 (0.48) |
| Years of regular cannabis use ** | 3.38 (3.30) | 0.40 (1.91) |
| Years of regular alcohol use * | 1.34 (2.90) | 3.35 (4.73) |
| Self-reported delinquency |  |  |
| Destruction/public order offence - lifetime | 60.7% | . |
| Property offence - lifetime | 83.9% | . |
| Aggression/violent offence - lifetime | 69.1% | . |
| Weapon offence - lifetime | 45.5% | . |
| Drug offence - lifetime | 64.3% | . |
| Self-reported police contact |  |  |
| none | . | 96.6% |
| Less than 1 contact per year | . | 3.4% |
| *Note.* WODC = WODC Self-reported Delinquency Questionnaire; BSSS = Brief Sensation Seeking Scale; BIS-11 = Barratt Impulsiveness Scale. * *p* < 0.05 measured with independent sample t-test ** *p* < 0.001 measured with independent sample t-test | | |
